# Ethnic differences in reading and mathematical test performance in primary schools in England

**DOI:** 10.23889/ijpds.v8i2.2355

**Published:** 2023-09-14

**Authors:** Alexey Bessudnov

**Affiliations:** 1 University of Exeter, Exeter, United Kingdom

## Objectives

This study investigates ethnic differences in Key Stage 2 (KS2) reading and mathematical test scores among primary school pupils in state schools in England between 2007 and 2018. The aim is to assess the performance across ethnic categories and examine its evolution over the course of the study period.

## Method

The analysis uses data from the National Pupil Database combining ethnicity information from school censuses with KS2 attainment data. KS2 reading and mathematical tests are taken by Year 6 pupils aged 10 to 11. Mean test scores are compared across ethnic categories, while the method of relative distribution is employed to evaluate performance in each ethnic category relative to the White British across the entire distribution of test scores in 2007 and 2018.

## Results

Between 2007 and 2018, the reading and maths test scores of British Bangladeshi, Black African, and Pakistani pupils improved relative to the White British group. In 2018, British Bangladeshi and Black African pupils performed at a similar or slightly higher level compared to their White British peers. The advantage in test scores in the two higher performing categories, British Indian and Chinese, further increased. Attainment in the other White category remained similar to the White British group. The test scores for the Black Caribbean and Mixed White and Black Caribbean categories tended to be concentrated in the lower part of the distribution. In 2018, the proportion of mixed and non-White pupils remained largely constant throughout the reading test score distribution, while in maths, a higher proportion of mixed and non-White pupils were found among high achievers compared to other parts of the distribution.

## Conclusion

From 2007 to 2018, KS2 test performance improved relative to the White British group for some ethnic categories, while it did not for others. This paper proposes potential explanations for these differences, which are related to the volume and characteristics of immigration to England. Further empirical testing is required in future research to substantiate these explanations.

